# Test and treat — community perspectives

**DOI:** 10.1186/1758-2652-13-S4-O14

**Published:** 2010-11-08

**Authors:** B Spire

**Affiliations:** 1 AIDES & INSERM U912, Marseille, France

## 

Antiretroviral treatment (ART) has dramatically changed the lives of people living with HIV/AIDS. Until recently and despite the benefits of antiretroviral therapy, people living within HIV still expressed their fears about treatment side-effects and about possible HIV transmission.

Accumulated evidence demonstrating the potent role of antiretroviral therapy in decreasing HIV transmission together with the availability of new generations of antiretroviral drugs with improved efficacy and tolerability has led to most HIV community leaders in France changing their attitudes in favor of adopting the "test and treat" approach. This change is also shared among leaders of HIV community-based associations in French speaking Africa. However, such an approach will not be possible without major changes in HIV policies. More than simply providing treatment availability it requires

i. a global policy against HIV discrimination and against HIV stigma in order to facilitate access to testing and treatment,

ii. the involvement and empowerment of those communities most concerned by HIV

iii. strong political leadership to both change the representation of HIV/AIDS in the general public and to implement innovative funding mechanisms.

It also implies the development of a strong international HIV policy for universal access to HIV care and prevention including respect of human rights, especially of sexual minorities, migrants and drug users. Today, the "test and treat" approach represents an important tool in curbing the HIV epidemic not only because it pushes political leaders to give top priority to HIV on their political agendas but also because it influences HIV policies which in turn encourage civil society to become directly involved in doing "with people" and not "for people".

